# Porous Silicon Nanoneedles Modulate Endocytosis to Deliver Biological Payloads

**DOI:** 10.1002/adma.201806788

**Published:** 2019-01-24

**Authors:** Sahana Gopal, Ciro Chiappini, Jelle Penders, Vincent Leonardo, Hyejeong Seong, Stephen Rothery, Yuri Korchev, Andrew Shevchuk, Molly M. Stevens

**Affiliations:** Department of Medicine Imperial College London Hammersmith Hospital Du Cane Road, London W12 0NN, UK; Department of Materials Royal School of Mines Prince Consort Road, London SW7 2AZ, UK; Department of Materials Royal School of Mines Prince Consort Road, London SW7 2AZ, UK; Facility for Imaging by Light Microscopy Imperial College London Sir Alexander Fleming Building Exhibition Road, London SW7 2BB, UK; Department of Medicine Imperial College London Hammersmith Hospital Du Cane Road, London W12 0NN, UK; Department of Materials Royal School of Mines Prince Consort Road, London SW7 2AZ, UK; Department of Bioengineering Royal School of Mines Prince Consort Road, London SW7 2AZ, UK; Institute of Biomedical Engineering Imperial College London Royal School of Mines Prince Consort Road, London SW7 2AZ, UK

**Keywords:** biointerface, drug delivery, endocytosis, nanoneedles, porous silicon

## Abstract

Owing to their ability to efficiently deliver biological cargo and sense the intracellular milieu, vertical arrays of high aspect ratio nanostructures, known as nanoneedles, are being developed as minimally invasive tools for cell manipulation. However, little is known of the mechanisms of cargo transfer across the cell membrane-nanoneedle interface. In particular, the contributions of membrane piercing, modulation of membrane permeability and endocytosis to cargo transfer remain largely unexplored. Here, combining state-of-the-art electron and scanning ion conductance microscopy with molecular biology techniques, it is shown that porous silicon nanoneedle arrays concurrently stimulate independent endocytic pathways which contribute to enhanced biomolecule delivery into human mesenchymal stem cells. Electron microscopy of the cell membrane at nanoneedle sites shows an intact lipid bilayer, accompanied by an accumulation of clathrin-coated pits and caveolae. Nanoneedles enhance the internalization of biomolecular markers of endocytosis, highlighting the concurrent activation of caveolae- and clathrin-mediated endocytosis, alongside macropinocytosis. These events contribute to the nanoneedle-mediated delivery (nanoinjection) of nucleic acids into human stem cells, which distribute across the cytosol and the endolysosomal system. This data extends the understanding of how nanoneedles modulate biological processes to mediate interaction with the intracellular space, providing indications for the rational design of improved cell-manipulation technologies.

Gaining access to the intracellular space with minimal toxicity is a key feature in developing efficient strategies for drug delivery[1] and intracellular sensing.[2] Vertical high-aspect ratio nanoneedles,[3–9] have demonstrated a broad versatility to efficiently sense[2] and deliver[10] to the cell interior. Successful delivery of exogenous materials in the form of nucleic acids,[4,5,9] proteins,[5] metabolites,[11] and cell-impermeable[12] nanoparticles by nanoneedle arrays, has now been independently demonstrated for a broad range of geometries, material compositions, and cell types. Moreover, the intimate interface established with the cell membrane has enabled nanoneedles to sense proteins,[13] metabolites,[14] and lipids[15] in the intracellular milieu as well as to stimulate and record action potentials of large arrays of individual excitable cells.[7,16,17] Nanoneedle arrays promise to be a safe and effective platform for nucleic acid delivery that compares favorably with microinjection and electroporation, thanks to its ease of use, high throughput, elevated biocompatibility, and efficient delivery.

Despite rapid advances that culminated with their first use for in situ gene therapy,[9] there is a need to improve the understanding of nanoneedle-mediated delivery in order to rationally design platforms for efficient clinical translation. Biomolecules delivered from nanoneedles display biological activity, highlighting an underlying mechanism that traffics them to their target site of action either in the cytosol (siRNA,[9] proteins,[5] or peptides[13]) or in the nucleus (plasmid DNA[9]). This evidence of efficient delivery led to the initial assumption that nanoneedles traversed the cell membrane to directly deliver biomolecules into the cytoplasm. Pioneering studies with atomic force microscopy (AFM) operated nanoneedles supported this assumption, showing systematic drops in the force–displacement curve during interaction with synthetic lipid bilayers[18,19] and membranes of cells in culture,[20–23] attributed to breaching of the cell membrane. The local quenching of green fluorescent protein (GFP) fluorescence upon nanoneedle delivery of membrane-impermeant Co^2+^ ions supported this view.[24]

Yet, direct evidence from microscopy analysis of the cell-nanoneedle interface, invariably showed an intact cell membrane around the nanostructures. Transmission electron microscopy (TEM) analysis of cells on lower aspect nanoneedles displayed a continuous cell membrane wrapping around all the pillars observed.[25] Similarly, studies of nanoneedles of different geometries and surface chemistries showed that at least 95% of the nanoneedles were enclosed within a membrane, with a determination not possible only in the case of four amino-silane-coated nanoneedles.[26] In agreement with this data, a more recent microscopy study showed cell membrane wrapping around nanoneedles of a range of lateral dimensions between 80 nm and 1 μm.[27] Further, all recent attempts using nanoneedles to sense intracellular potentials in excitable cells, agree on the need for an initial electroporation or optoporation in order to gain access to the cytosol, and that such access is only temporary, albeit much longer than conventional clamping methods.[7,16,27,28] Raman spectroscopy also allowed identifying the initial presence of a membrane wrapping around SERS active hollow nanoneedles, its disruption upon poration and the ensuing resealing process, that led to changes in membrane composition.[15] Overall these findings indicate that a simple membrane penetration model does not fully describe the uniqueness and complexity of the cell-nanoneedle interface, calling for more in-depth investigations of its nature, and of the mechanisms that enable intracellular delivery.

For instance, we must consider the crucial role played by endocytosis in trafficking cargo from the cell membrane, a dynamic and complex system that is highly responsive to external signals, both mechanical and biochemical. Indeed, deforming the cell membrane, as nanoneedles do, can modulate the local composition of lipids and proteins,[29] inducing the accumulation of intracellular scaffolding structures that initiate clathrin or caveolae-mediated endocytosis.[30] High-aspect nanostructures that locally deform the membrane can accumulate scaffolding proteins and endocytic pits.[31–33] However, the contribution of endocytosis to biomolecule delivery from nanoneedles, and its role on determining the fate of the payload still needs to be clarified.

In this study, we investigate the interface between the cell membrane and recently developed porous silicon nanoneedles[9,12] in relation to their capacity to deliver biological payloads in human mesenchymal stem cells (hMSCs). In particular, we assess the ability of porous silicon nanoneedles to deliver biological cargo via endocytosis, while negotiating the cell membrane using a variety of state-of-the-art microscopy techniques. Moreover, we study the fate of the delivered pathway-specific cargo within the cytoplasm, highlighting the significant role of endocytic pathways in nanoneedle-mediated delivery (nanoinjection). Finally, the significant endolysosomal trafficking of nanoneedle-injected siRNA highlights a role for endocytosis in nucleic acid uptake, while concurrently validating its cytosolic delivery and biological activity by gene silencing. These results provide fundamental insight into the contribution of topographic stimuli on intracellular delivery in nonendocytic cells, which has implications for the design of next generation stem cell manipulation strategies involving intracellular biomolecule delivery and interrogation.

In order to assess the membrane response to interfacing with porous silicon nanoneedles, the apical membrane morphology of hMSCs was visualized on nanoneedle arrays and flat silicon substrates using a combination of electron and scanning ion conductance microscopy (SICM) techniques. SICM enabled label-free, noninvasive mapping of the topography of the apical cell membrane in its native state[34–36] (Figure S1a, Supporting Information). After 6 h of culture, the apical surface of hMSCs on nanoneedles showed numerous protrusions in random orientations akin to dorsal membrane ruffles seen in cells actively participating in macropinocytosis (MP)[37] and remodeling their actin cytoskeleton[38] (Figure 1a and Figure S1b,c, Supporting Information). It is in these regions that actin-related protein 2/3 (ARP2/3) complexes and vesicle scission proteins such as dynamins are known to localize.[39] Membrane ruffling led to a 1.8-2-fold increase in surface roughness (*R_r_*_ms_ and *R*_a_) of the apical membrane of cells interfaced with nanoneedles compared to those cultured on flat silicon wafers (FSW) (Figure 1b,c). To characterize the morphology of the basal membrane in contact with the nanoneedles, we used a recently developed focused ion beam scanning electron microscopy (FIB-SEM) approach[9,40] for in situ slice-and-view[40,41] (Figure 1d and Figure S2a–e, Supporting Information), alongside analysis of resin-embedded sections by TEM (Figure 1e and Figure S3, Supporting Information). FIB-SEM showed the basal membrane of hMSCs wrapping around individual nanoneedles without discontinuities evident at 20 nm resolution (Figure S2d,e, Supporting Information). Higher resolution TEM micrographs of FIB-milled sections showed extreme vicinity (in the order of nanometers) between the nanoneedles and the membrane at the interface and confirmed membrane continuity at 4 nm resolution (Figure 1e).

In the absence of visible membrane penetration or discontinuities for long-term interfacing, and given the observed membrane ruffling, endocytosis represents a viable mechanism that can contribute to payload delivery. Immunofluorescence analysis highlighted the localization of caveolin-1 (CAV-1, Figure 2a–d), a key protein involved in caveolae-mediated endocytosis (CavME) and clathrin light chain (CLC, Figure 2e–h), a clathrin coat protein for clathrin-mediated endocytosis (CME) after 6 h of interfacing. Both CAV-1 and CLC colocalized with nanoneedles at the basal membrane, as they clustered with periodic intensity. Fourier transform analysis confirmed that the period (1.967 +/− 0.05 μm) matched the nanoneedle spacing (2 μm) (Figure 2d,h). The same proteins at the apical membrane were unaffected by the nanoneedles and we observed no periodicity basally or apically for hMSCs on FSW (Figure 2c,g). Moreover, the total protein expression levels of CAV-1 and CLC did not increase on nanoneedles suggesting that this response is local to the membrane-nanoneedle interface (Figure S4, Supporting Information). Indeed, FIB-SEM analysis confirmed the assembly of these endocytic proteins into spherical-coated pits and flask shaped invaginations in the size range of clathrin-coated pits (CCP) and caveolae (Figure 2i and Figure S2e, Supporting Information), and their preferential accumulation near the tips and sides of the nanoneedles (Figure 2j,k). Previous reports of cells cultured on nanopillars suggest that caveolae are not sensitive to membrane curvature.[31] However electron microscopy, confocal imaging, and delivery of caveolae-specific cargo in our system indicate that caveolae are key players during interfacing, most likely through their ability to sense membrane tension rather than curvature in a mechanically stressed environment.[42] These data indicate that nanoneedles induce local membrane deformation that accumulates functional endocytic vesicles.

Given the accumulation of endocytic vesicles at the nanoneedle interface and the membrane ruffling, we investigated whether and to what extent nanoneedles stimulated specific endocytic pathways. We assessed CME by Transferrin (Tfn) uptake, CavME by internalization of Cholera toxin B-subunit (CTxB) and MP by uptake of Dextran (Dex) chains in the size range of 10 to 70 kDa (Figure 3a, clockwise). We measured the adsorption efficiency of Tfn, CTxB, and Dex to find that these cargoes adsorbed in similar amounts on nanoneedles and FSW suggesting that the maximal loading of the nanoneedle substrate is dependent on the size and charge of the cargo, nanoneedle pore size and surface charge rather than total surface area (Figure S5, Supporting Information). After 24 h, Tfn, CTxB and Dex 10, 40, and 70 kDa were all internalized more efficiently by cells cultured on nanoneedle arrays compared to FSW as indicated by the significant increase in the percentage of positive cells for each of the cargo (Figure 3a and Figure S7–S11, Supporting Information).

In order to assess the proportion of endocytic cargo that enters the endolysosomal system during nanoneedle-mediated delivery, we assessed biomolecules known to internalize through specific endocytic pathways for their colocalization with early endosomes and lysosomes. In addition, we determined the colocalization of Tfn (known to enter by CME) against CLC, and the colocalization of CTxB (known to enter by CavME) with Cav-1 at 24 h. At this point the nanoneedles have degraded[9] and no array-like pattern of Cav-1 and CLC was observed. Tfn colocalized with CLC (46 ± 16%) and lysosome-associated membrane protein 1 (LAMP1) (49 ± 20%) and Early Endosome Antigen 1 (EEA1, 33 ± 12%), indicating that Tfn may be recycled to the cell surface (associated with CLC at cell surface) and trafficked into lysosomes (Figure 3b). The extent of colocalization for CME and CavME cargoes with endolysosomal compartments and carrier proteins was highly variable. For instance, the colocalization of CTxB with its carrier Cav-1 and with EEA1 ranged from below 20% in some cases to above 90% in others (Figure 3c, graph panel). This variation suggests that nanoneedles enable sustained delivery of CTxB resulting in cargo at different stages of trafficking along each pathway. CTxB however colocalized highly with LAMP1, suggesting that CavME stimulated by nanoneedles accumulated within lysosomes. Unsurprisingly, Dex showed highly consistent degrees of colocalization with endosomes and lysosomes (20–30%), possibly owing to the fact that macropinocytosis occurs from the apical cell surface and relies on release of fluid phase markers into the surrounding media for internalization rather than from the basal cell surface in contact with the nanoneedles (Figure 3d). Lastly, we assessed the fate of each of these cargoes within the endolysosomal system by combining their colocalization with respective carriers, endosomes and lysosomes to find that CTxB was trafficked into the endolysosomal system to the greatest extent (65 ± 20%), followed by Tfn (45 ± 17%), and Dex (38 ± 11%) (Figure 3e). This indicates that specific endocytic pathways are activated by nanoneedles, and account for a significant fraction, but not the entirety, of the total delivery of cargo-specific payload.

If endocytosis is the key mechanism of internalization by nanoinjection, the entrapment of biofunctional payloads within the endolysosomal system could hamper their functionality. To test this hypothesis we investigated the trafficking of nanoinjected siRNA targeting GAPDH and probed its biological activity, which relies on the colocalization of nondegraded siRNA with the RNA-induced silencing complex (RISC) in the cytosol.[43] Indeed, nanoinjection significantly improved intracellular delivery of siRNA-GAPDH (Figure 4a). After 24 h of interfacing, the siRNA localized in the cytoplasm of hMSCs with a considerable amount of punctate signal, typically in the perinuclear region (Figure 4b). siRNA could be seen colocalizing with Cav-1, CLC, EEA1, and LAMP1 to different extents (Figure 4c–f), and its colocalization with these components was quantified. An average of 45 ± 13% of siRNAs colocalized with Cav-1, 34 ± 9% of siRNAs colocalized with CLC, 44 ± 14% of siRNA with EEA1 and 40 ± 13% of the siRNAs were entrapped within LAMP1 positive lysosomes, indicating that a substantial percentage of nucleic acids enter the endolysosomal pathway (Figure 4g). By considering the colocalization of siRNA with the combined signal of EEA1, Lamp1, CLC, and Cav-1, 62 ± 16% of siRNA was trafficked into the endolysosomal pathway (Figure 4g). Nevertheless, nanoinjection of siRNA-GAPDH induced a statistically significant 43 ± 14% reduction in GAPDH expression in hMSCs compared to control in each experiment (*N* = 3, *p < 0.05, Student’s paired *t*-test, Table S1, Supporting Information), indicating that a proportion of the remaining 38% of nanoinjected siRNAs are trafficked outside of endolysosomal pathway and can mediate biological functions in the cytosol.

Overall, our results demonstrate that nanoneedles improve the internalization of pathway-specific payloads and nucleic acids. This delivery is at least partly mediated by endocytic processes which are upregulated selectively at the cell-nanoneedle interface, leading to the endolysosomal localization of large proportions of the payloads. Nonetheless, a significant fraction of each payload (38% for siRNA) is trafficked alternatively and retains biological function in the cytosol. The active cytosolic fraction of payload could result from endosomal escape or concurrent delivery mechanisms that bypass the endolysosomal system. In the literature, studies of nanoneedle-mediated gene therapy have shown transfection efficiencies widely varying between 0% and > 90% depending on nanoneedle properties and cell type.[3–5,9,44] The engagement of specific endocytic pathways can regulate payload fate and could play a significant role in the broad differences observed. This study highlights that these aspects should be investigated when developing nanoinjection strategies in order to optimize their efficiency.

## Experimental Section

Available in Supporting Information.

## Supplementary Material

Supporting Information is available from the Wiley Online Library or from the author.

Supplementary Information

## Figures and Tables

**Figure 1:**
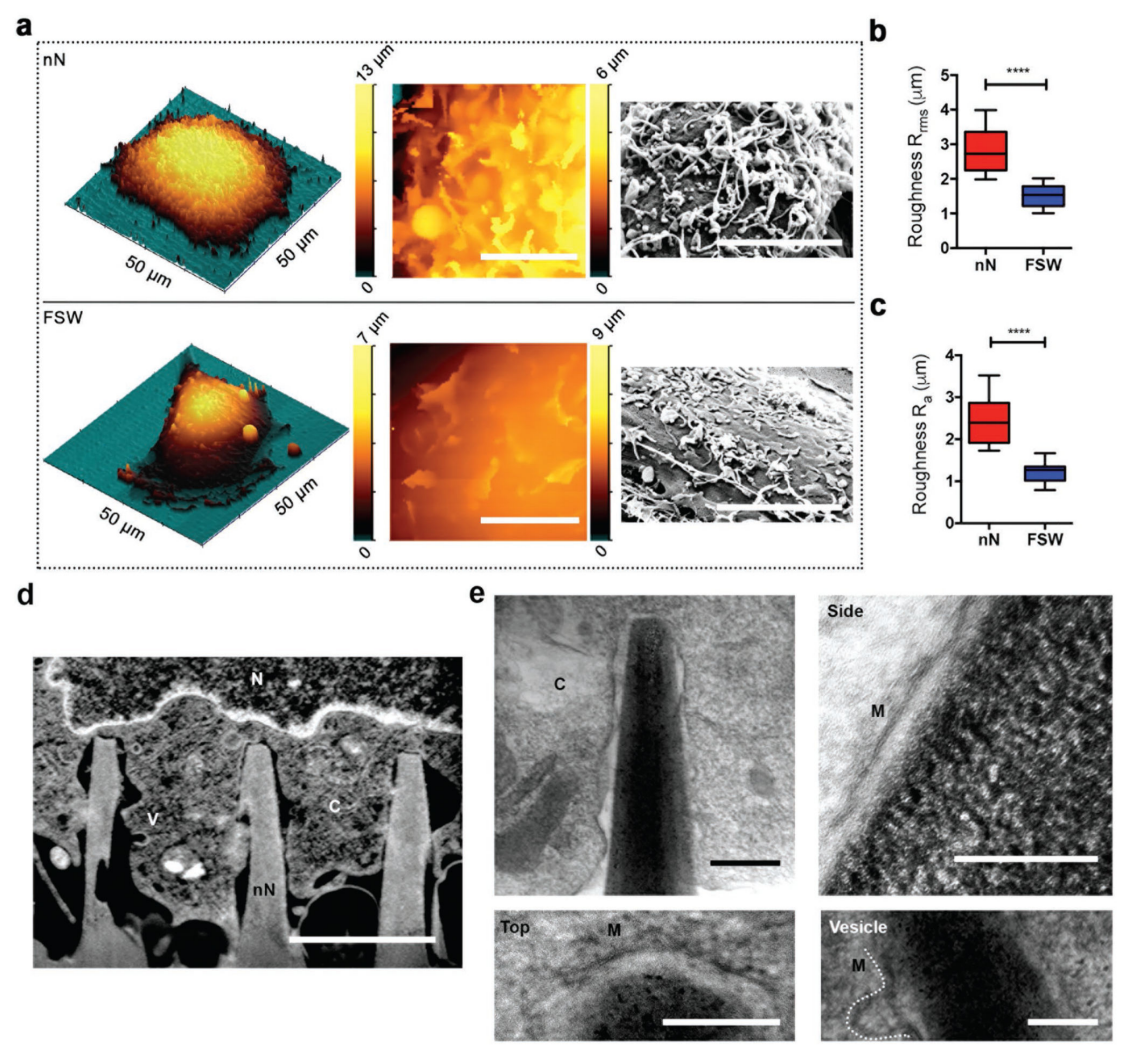
Cell membrane response to nanoneedle (nN) interfacing. a) Nanoneedle interfacing induces membrane ruffling. 3D SICM image of an hMSC cultured on nanoneedles (top) or FSW (bottom) for 6 h (left). A zoomed-in 2D SICM scan (10 × 10 μm) (middle) and SEM image of the apical membrane (right). Scale bars = 5 μm. b,c) Nanoneedle interfacing increases membrane roughness. Surface roughness (b) *R*_rms_ and (c) *R*_a_ of apical membrane of hMSCs on nanoneedles compared to FSW measured by SICM. Box plot shows center line as median, first and third quartile data range, and whiskers to minimum and maximum. ****p < 0.0001 (two-tailed unpaired Student's *t*-test), *n* = 8 cells for nN and *n* = 11 cells for FSW. d,e) Cell membrane integrity is observed at the nanoneedle interface. C = cytosol, V = vesicle, N = nucleus, M = membrane. (d) Representative FIB-SEM image of an orthogonal cross section of an hMSC on nanoneedles after 6 h of interfacing. Scale bar = 2 μm. (e) TEM of FIB lift-out thin sections of the hMSC-nanoneedle interface. Clockwise: overview of a representative nanoneedle; nanoneedle side; vesicle located at the side of a nanoneedle; nanoneedle top. Scale bar = 200 nm.

**Figure 2:**
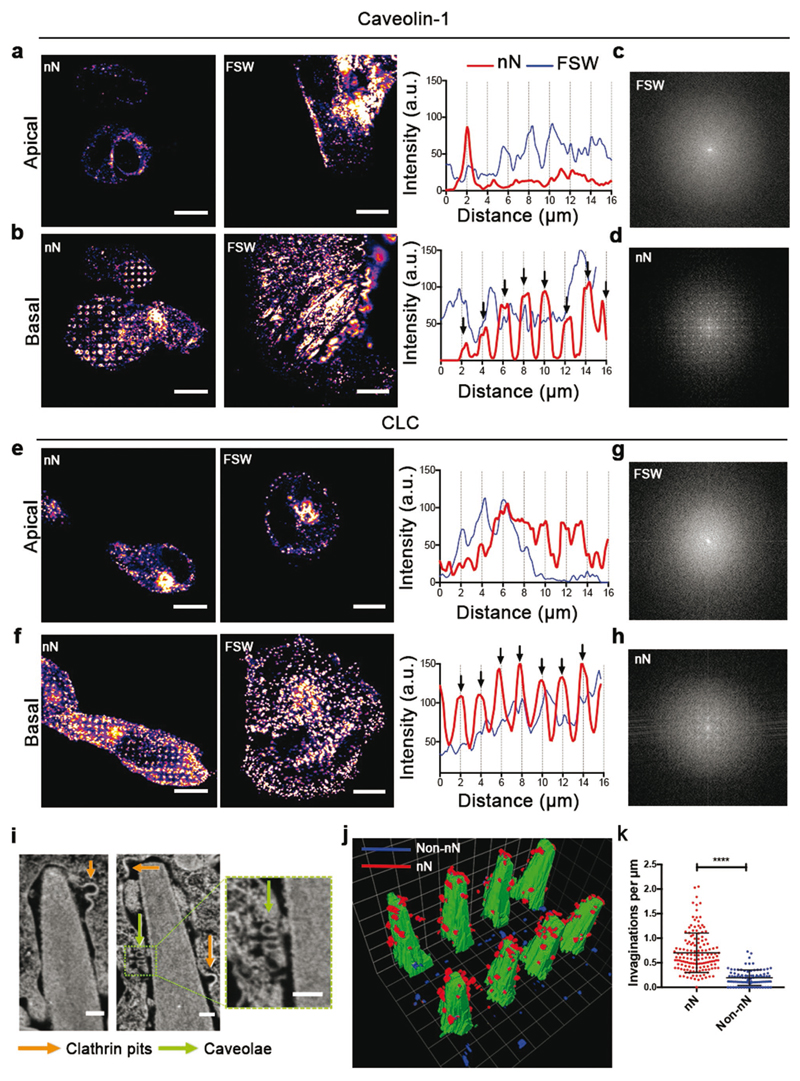
Nanoneedles locally activate endocytosis. a–d) Caveolin-1 (Cav-1) accumulates around nanoneedles. a,b) Confocal fluorescence images of caveolin-1 after 6 h in the (a) apical and (b) basal membrane of hMSCs cultured on nanoneedles or FSW and their respective line intensity profiles over the cells. Arrows in line intensity plots indicate 2 μm intervals matching the distance between individual nanoneedles. c,d) Upon interfacing, Cav-1 at the basal membrane acquires the same periodicity as the nanoneedles as assessed by Fourier transform analysis of the basal surface of hMSCs in panels (a) and (b). e,f) Clathrin accumulates around nanoneedles. Confocal fluorescence images of CLC after 6 h in the (e) apical and (f) basal membrane of hMSCs cultured on nanoneedles or FSW and their respective intensity profiles along the dashed lines. g,h) Upon interfacing, clathrin at the basal membrane acquires the same periodicity as nanoneedles as assessed by Fourier transform analysis of the basal surface of hMSCs in panels e and f. Scale bars = 10 μm. i–k) Endocytic vesicles accumulate around nanoneedles. (i) FIB-SEM image interface showing two classes of endocytic vesicles accumulating around nanoneedles: clathrin pits (orange arrows) and caveolae (green arrows). Scale bars = 100 nm. (j) 3D reconstruction of the cell-nanoneedle interface over two consecutive rows of nanoneedles highlighting vesicular structures present in the membrane at nanoneedle (red) and non-nanoneedle (blue) locations. (k) Quantification of vesicular invaginations in the membrane from FIB-SEM data at nanoneedle and non-nanoneedle locations. Plot shows mean ± S.D., *N* = 4, *n* = 11 (cells), ****p < 0.0001 (two-tailed unpaired Student's *t*-test).

**Figure 3:**
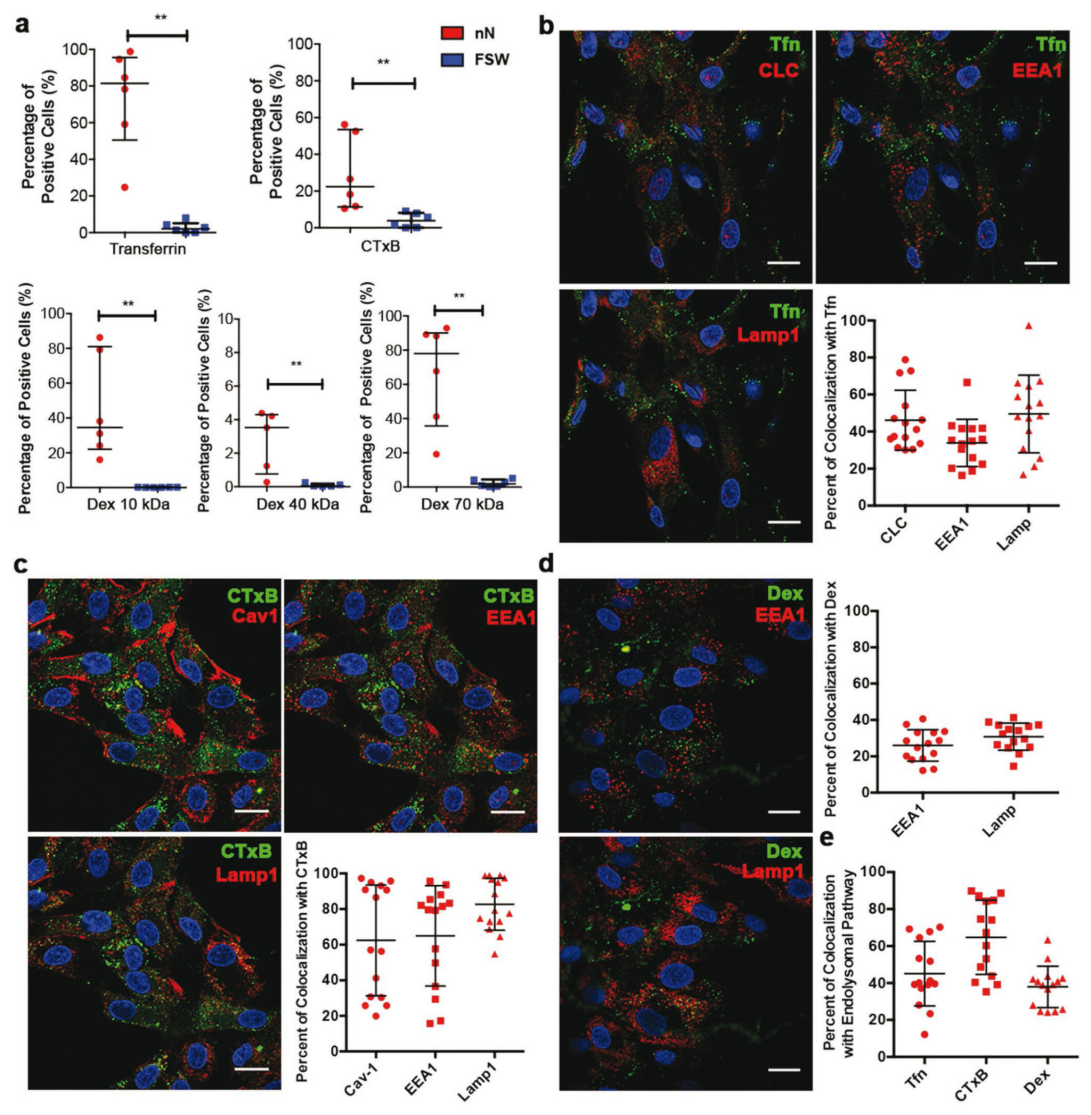
Nanoinjection enhances the uptake of pathway-specific cargo, which localizes within the endolysosomal system. a) Nanoinjection enhances uptake of Tfn, CTxB, and Dex of different sizes. Flow cytometry analysis showing the percentage of positive cells successfully internalizing pathway specific payloads by nanoinjection or FSW delivery at 24 h. Clockwise – Tfn, a clathrin-mediated endocytosis cargo, CTxB, a caveolae-specific cargo, and Dex 10, 40, 70 kDa, Macropinocytosis-specific cargo. Data presented as median with interquartile range. *N* = 3, *n* = 2 **p = 0.0022 for Tfn, CTxB, Dex 10, Dex 70 kDa and **p = 0.0079 for Dex 40 kDa (Mann–Whitney test). b) Tfn fate. Representative confocal images of a plane above the nanoneedles and quantification of Tfn (green) colocalization with CLC, EEA1 and LAMP1 at 24 h. c) Cholera Toxin fate. Representative confocal images of a plane above the nanoneedles and quantification of CTxB colocalization with Cav-1, EEA1 and LAMP1 at 24 h. d) Dex fate. Representative confocal images of a plane above the nanoneedles and quantification of Dex 10 kDa colocalization with EEA1 and LAMP1 at 24 h. Quantified data represented scatter dot plots with bars representing mean ± S.D., *N* = 1, *n* = 3 biological replicates, at least five images per *n*. Scale bars = 20 μm. e) Quantification of Tfn, CTxB and Dex 10 kDa localization with their pathway-specific endocytosis carriers and trafficking components (Tfn with CLC, EEA1 and LAMP1, CTxB with Cav-1, EEA1 and LAMP1 and Dex10 with EEA1 and LAMP1). Quantified data presented as scatter dot plots with bars representing mean ± S.D., *N* = 1, *n* = 3 biological replicates, at least five images per *n*.

**Figure 4:**
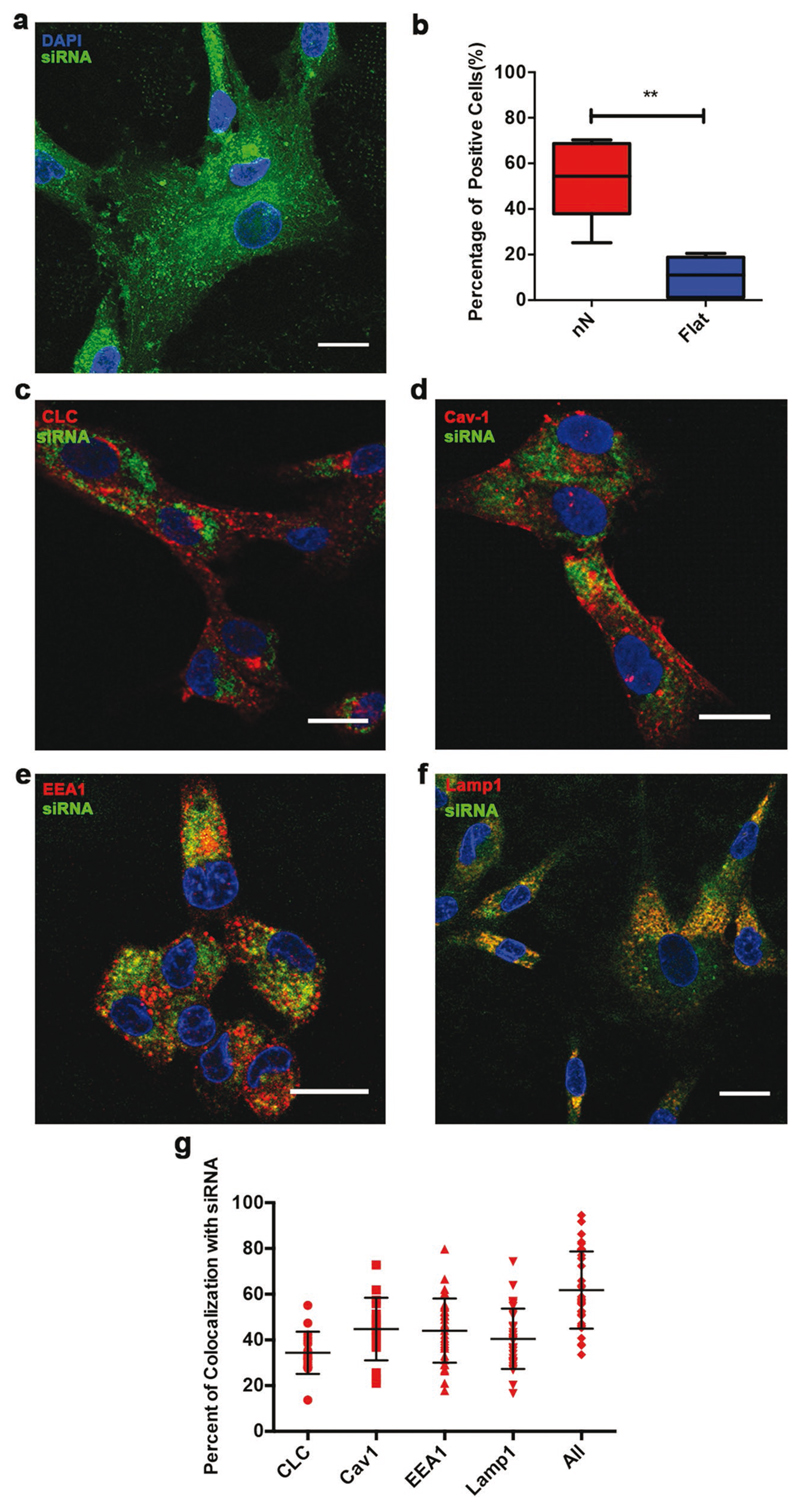
Nanoinjected siRNA partly localizes across the endolysosomes while retaining cytosolic activity. a,b) Nanoinjection enhances siRNA delivery. (a) Maximal Z-stack projection of hMSCs above the nanoneedles showing fluorescently labeled Cy3-siRNA-GAPDH (green) in the cell after 24 h of interfacing. (b) Flow cytometry data of Cy3-siRNA uptake in hMSCs mediated by nanoneedles compared to FSW after 24 h. Box plot shows center line as median, first and third quartile data range, and whiskers to minimum and maximum. **p = 0.0022 (Mann–Whitney test), *N* = 4, *n* = 2. c–f) Representative confocal images of Cy3-siRNA colocalization with (c) CLC, (d) Cav-1, (e) EEA1, and (f) LAMP1. Scale bars = 20 μm. g) Colocalization of endocytic carrier proteins (CLC, Cav-1), endosomes (EEA1) and late endosomes/ lysosomes (LAMP1) and their combination (All) with Cy3-siRNA. Values reported as aligned scatter plot of percentages of Cy3-siRNA pixels overlapping with indicated components of the endolysosomal system (Mander's coefficient). Lines represent mean ± S.D. *N* = 3, *n* = 2 for LAMP1, EEA1, *N* = 3, *n* = 1 for CLC, Cav-1. —Five to ten images per sample.
