# Supplementary material for: Porous Silicon Nanoneedles Modulate Endocytosis to Deliver Biological Payloads
Source: Adv Mater. Author manuscript; Available in PMC 2019 Jul 2. (PMC6606440; doi:10.1002/adma.201806788)
Supplement: Supplementary Information [file EMS83152-supplement-Supplementary_Information.docx]

Supporting Information

Title Porous silicon nanoneedles modulate endocytosis to deliver biological payloads

Author(s), and Corresponding Author(s)*

Sahana Gopal^1,2^, Ciro Chiappini^3^, Jelle Penders^2^, Vincent Leonardo^2^, Hyejeong Seong^2^ Stephen Rothery^4^, Yuri Korchev^1^, Andrew Shevchuk^1^, Molly M. Stevens^2,5^*

Experimental Section

Antibodies and reagents

Anti-Caveolin-1 was purchased from Abcam (ab2910), Anti-clathrin light chain was purchased from Novus Biologicals (NBP2-14913), Anti-EEA1 (C45B10), Anti-LAMP1 (D4O1S) were purchased from Cell Signaling Technologies. Anti-Cy3-PE was purchased from Santa Cruz Biotechnologies. Transferrin-Alexa488, Cholera Toxin B subunit-Alexa488, Albumin-AlexaFluor®488, Dextran-TexasRed (10kDa, 40kDa and 70kDa) and GAPDH siRNA-Cy3 were purchased from ThermoFisher Scientific.

Nanoneedle fabrication and surface treatment

Nanoneedles were fabricated according to our established protocols.^[1]^ We deposited a 120 nm layer of low stress silicon nitride over 0.01-0.02 Ω cm p-type 100 mm Si wafer. We patterned 0.6 µm clear field dots with 2 µm pitch using contact UV photolithography uniformly through the wafer using NR9-250P photoresist (Futurrex, USA) in an MA6 mask aligner (K. Suss, Germany). The pattern was transferred into the silicon nitride layer by reactive ion etching in CF_4_ gas. Electroless deposition of 0.02 M AgNO_3_ salt in a 10% aqueous solution of HF for 2 minutes generated dendritic Ag nanoparticles selectively on the exposed silicon. Metal assisted chemical etching in 1% H_2_O_2_, 10% HF aqueous solution for 8.5 minutes generated porous silicon pillars underneath the silver nitride dots with interspersed porous silicon grass. The grass was removed, and the pillars shaped into cones by reactive ion etching in SF_6_ gas. The wafer was diced in 8 x 8 mm dies for further use. Nanoneedle and flat silicon wafer (FSW) substrates were treated with oxygen plasma for 10-20 minutes (PlasmaPrep II, Gala Instrumente, Germany). Nanoneedle and FSW substrates were treated with oxygen plasma for 10-20 minutes (PlasmaPrep II, Gala Instrumente, Germany).

Cell culture

Human mesenchymal stem cells (hMSC) were purchased from Lonza and cultured in MSCGM-CD (Lonza) or MSC Basal Media (MSCBM) (Lonza) with supplementation according to the manufacturer’s instructions. When ~80% confluent, hMSCs were detached with 0.05% (v/v) trypsin-EDTA (Invitrogen) and seeded onto FSW or nanoneedles substrates placed in a 24 well plate in Minimum Essential Media alpha (Gibco) with 10% (v/v) hMSC-graded foetal bovine serum (Gibco) and 1% (v/v) Penicillin/Streptomycin (Gibco). HMSCs were cultured on FSW and nanoneedle substrates at either passage 4 or passage 5 and seeded at a density of 20,000 cells per square cm unless otherwise specified.

Scanning ion conductance microscopy

SICM images of live hMSC cells were acquired in hopping probe scanning mode using a home-built microscope described previously.^[2]^ Pipettes of approximately 100 nm were pulled from borosilicate glass (O.D. = 1 mm, I.D. = 0.5 mm, Intracel, Cambridge, UK) using a P-2000 laser puller (Sutter Instruments). Ion current measurements were conducted using Axopatch 200B amplifiers (Molecular Devices). Pipettes were filled with sterile filtered PBS for each experiment. For imaging, a bias potential of 200 mV was used. Ion current traces were tracked using pClamp10 (Molecular Devices). Cells cultured on nanoneedles and FSW for 6 hours were fixed in 4% (v/v) methanol-free paraformaldehyde solution (PFA, EM Sciences) for 15 minutes, washed with PBS and placed in 35 mm dishes in PBS. The pipette was immersed, current detected and approached over the sample using a set point of 0.3-0.4% drop in current. Cells were located by observing the z-retraction of the piezo when an area was scanned in x and y directions. A quick low-resolution scan was conducted to confirm the presence of the cell. The area containing the cell was selected and scanned at high resolution. Due to the high aspect ratio of the nanoneedle substrate, a pre-scan hop size of 10 μm was used when imaging cells on nanoneedles. Images were analyzed using a home-written SICM Image viewer and surface roughness values were obtained using free, open source Gwyddion software for scanning probe microscopy data visualization and analysis (www.gwyddion.net).

Scanning electron microscopy

hMSCs cultured on nanoneedles or FSW were fixed in 2.5% (v/v) glutaraldehyde (GA, EM Sciences) for 30 minutes at room temperature. Samples were treated with 1% (v/v) OsO_4_ (EM Sciences) for 1 hour in 0.1 M sodium cacodylate buffer. Samples were washed twice in double distilled water for 5 minutes and dehydrated with a series of graded ethanol concentrations (20, 30, 50, 70, 80, 90%) and finally in 100% ethanol 4 times for 5 minutes each. Samples were treated with hexamethyldisilazane (Sigma) for 5 minutes and air dried after which they were mounted on aluminium stubs using carbon tape and sputter-coated with 10 nm of chromium (Quorum 150T). Samples were visualized using a Zeiss Sigma300 SEM at an accelerating voltage of 5 kV and a working distance of 5 mm and secondary ion detector.

FIB-SEM sample preparation

hMSCs cultured on nanoneedles or FSW were fixed with GA, treated with OsO_4_ and washed as above. Samples were treated with 1% (v/v) tannic acid (Sigma) in water for 1 hour and washed with water. Samples were stained with 1% (v/v) uranyl acetate solution for a minimum of 2.5 hours in the dark and proceeded to dehydration in ethanol as mentioned above. Resin embedding was conducted with epoxy resin embedding kit (Epon812, Sigma) at 3:1, 2:1, 1:1, 1:2 (v/v) of ethanol to resin for 2.5-3 hours each. Pure resin was infiltrated the following day for 3 hours, twice. Excess resin was washed off with ethanol twice, with air drying the sample between washes. Samples were left to polymerize for 48 hours at 60° C.

FIB-SEM imaging and analysis

Samples were sputtered with 10 nm chromium or 20 nm gold and imaged using Auriga CrossBeam Workstation (Zeiss). Cells on nanoneedles were located using the SEM and the stage was tilted to 54° and a tilt correction of 36° was applied. The same cell was located using the FIB beam at a working distance of 5 mm. For 3D reconstructions, an imaging interval yielding 30 nm sections was used by milling with 1 nA:30 kV milling current. For vesicle number analysis, the sample was milled at 1 nA:30 kV until a row of nanoneedles appeared and a single image obtained at the estimated tallest part of the nanoneedles-membrane interface. Non-nanoneedles locations were also imaged in the same cell as a control. SEM images were obtained with an accelerating voltage of 1.6 kV using a backscattered electron detector.

For 3D reconstruction, sequential images were aligned manually using Fiji and Amira (FEI). Images were manually segmented and reconstructed for nanoneedle, cell membrane and vesicle at nanoneedle locations and non-nanoneedle locations. For quantification of vesicle numbers, the length of the basal membrane in each image was calculated using the Fiji plug-in Simple Neurite Tracer. The number of vesicles in each image at nanoneedle and non-nanoneedle locations were counted manually with the point tool in Fiji.

Lift-out and TEM

Lamellae of hMSCs seeded on nanoneedles were prepared for TEM/STEM imaging by FIB-SEM lift-out using a Helios Nanolab 600 (FEI, Eindhoven, The Netherlands) equipped with a Ga^+^ ion beam and a micromanipulator (Omniprobe, Oxford Instruments plc, Oxfordshire, United Kingdom). SEM imaging was performed at 2 kV and 0.17 nA and FIB milling at 30 kV with currents ranging from 28 pA to 2.8 nA, FIB polishing was performed down to 5 kV and 8 pA. A cell of interest was located by SEM imaging, showing normal morphology and proper preservation (Figure S3a). The sample was oriented to have the nanoneedle rows perpendicular to the FIB direction. First a rough trench was milled in the front of the cell at 2.8 nA, then the region of interest was approached by milling at 0.92 nA while simultaneously imaging in SEM mode. After ensuring the cell staining and ultrastructure preservation was adequate, milling was continued until the start of the row of nanoneedles included in the region of interest (Figure S3b). Platinum was deposited on top (1-1.5 μm wide) by Ga^+^ assisted GIS at 93 pA – 0.28 nA, up to a thickness of 1-2 μm. Further trenches were milled behind the region of interest and on the sides, freeing the lamella except for attachment to the base (Figure S3c). The lamella was approached with the micromanipulator needle and attached by platinum deposition. The base of the lamella was milled loose and for structural stability of the lamella up to 4 micron of silicon substrate was included in the lift-out (Figure S3d). The middle post of a 3-post copper lift-out grid (Omniprobe, EM Sciences, Hatfield, PA, USA) was approached with the micromanipulator and the lamella was attached to the post by further platinum deposition, after which the micromanipulator was cut loose (Figure S3e). The lamella was thinned to electron transparency, 100-120 nm, by FIB milling equally on both front and back of the lamella ensuring the apex of the nanoneedles is in the centre of the lamella (pre-thinning Figure S3f, post-thinning Figure S3g side view, Figure S3h front view).

Immunofluoresence staining, imaging and analysis

hMSCs on nanoneedles and FSW were fixed with 4% (v/v) PFA (EM Sciences) for 15 minutes in PBS. Samples were washed in PBS and treated with 0.25% (v/v) Triton X-100 for 5 minutes. Samples were washed with PBS and incubated with 5% (v/v) normal serum of the same species as the secondary antibody for 1 hour at room temperature. Primary antibodies to caveolin-1 (1:300), clathrin light chain (1:300), EEA1 (1:200) and LAMP1 (1:200) were allowed to incubate overnight at 4° C in 0.1% (w/v) BSA in PBS. For LAMP1 staining, PFA fixed samples were treated with methanol for 10 minutes at -20° C. For double immunofluorescence staining with multiple primary antibodies of the same species, primary antibodies to caveolin-1 and clathrin light chain were applied as mentioned and samples were washed in PBS. Samples were blocked using goat anti-rabbit Fab’ fragment (Jackson Immunolabs). Secondary fluorescent anti-goat antibodies were applied to detect caveolin-1 and clathrin. Following this, antibodies to EEA1 and LAMP1 were applied to these samples for detection with secondary fluorescent antibodies (Control experiment – Supplementary Figure 6).

Samples were washed 3 times for 5 minutes in PBS and secondary antibodies were applied for 1 hour at room temperature in the dark. Samples were washed extensively in PBS, flipped upside down into chamber slides (Ibidi) containing Vectashield (Vector Labs) for imaging by confocal microscopy. Images were obtained using a Leica SP5 inverted microscope using a 63X oil immersion objective.

For colocalization analysis, multi-channel images of 16-bit and 1024 x 786 pixel resolution were imported into Volocity Software (6.3, Perkin-Elmer) using automatic thresholding using the Costes’ method. This approach finds the best linear regression of the two channels of interest intensities over all the pixels, and then the thresholds are moved along the regression line until Pearson’s correlation for pixels below threshold is less than or equal to 0. In this way, all intensity values below the threshold (i.e cytosolic) were excluded from the analysis. Mander’s coefficient of overlap was selected and converted to a percentage for statistical analysis. For endolysomal pathway colocalization analysis, multi-channel images were overlaid in a single channel and analysed for colocalization with delivered payload as mentioned above.

Nanoneedle-mediated biomolecule delivery

Fluorescently labelled Transferrin (25 μg/ml), Albumin (40 μg/ml), Cholera Toxin (5 μg/ml), Dextrans (1 mg/ml) (10 kDa, 40 kDa and 70 kDa) and GAPDH-siRNA (1 µM) were each reconstituted in a buffer containing 0.25 M glycine and 400 mM KCl, pH adjusted to 5.0. Nanoneedle and FSW chips were plasma and UV treated prior to loading cargo. For siRNA delivery, chips were pre-treated with 2 M HCl for 10 minutes after plasma and UV treatment followed by rinsing in distilled water and air drying. 10 μl of each solution was adsorbed for 45 minutes in a humidified chamber in the dark. Chips were washed once with the buffer and air-dried. Chips loaded with siRNA were washed further with Buffer RPE (Qiagen) and air-dried. Cells were cultured on loaded substrates for 24 hours after which they were fixed in 4% (v/v) PFA or assessed for uptake by flow cytometry. For siRNA experiments, hMSCs were thawed from frozen, cultured to 70 % confluence and seeded at P4.

For assessing the uptake from cargo-loaded nanoneedles and FSW substrates, hMSCs were dry seeded by applying a 50 μl cell suspension containing 80-100,000 cells that stayed only on the chip and did not flood the well plate. After 2 hours, after the cells had attached to the substrates, the media was gently topped up to an optimum volume. This ensured that all cells seeded remained on the substrates and did not attach to the bottom of the wells.

Flow cytometry

Media was aspirated and chips were transferred to a new well plate and washed with PBS. Cells were detached from substrates with trypsin and centrifuged at 300 g for 7 minutes at 4° C. Cells were resuspended in PBS and centrifuged once again, after which they were resuspended in 200 μl of Cell Staining Buffer (BioLegend). Samples were measured with a BD LSRFORTESSA (BD) cell analyzer equipped with 488 nm and 561 nm lasers and 530/30 582/15 and 610/20 filters. Raw data were analyzed with FlowJo and equal population gates were applied in order to allow direct comparison between experimental groups.

Quantitative real time polymerase chain reaction (qRT-PCR)

Cells on nanoneedle and FSW substrates were treated with Trizol reagent (Life Technologies), mixed with chloroform (1:5, Chloroform:Trizol) and separated by centrifugation for 15 min at 4° C at 12,000g. RNA was isolated from the aqueous phase after the centrifugation using Direct-zol RNA MiniPrep Kit (Zymo Research) based on the manufacturer’s instructions. cDNA was synthesized using High Capacity cDNA Reverse Transcription Kits (Applied Bioscience) according to the manufacturer's instructions. qRT-PCR was performed with the PowerUP SYBR Green Master Mix (Applied Biosystems), with 2.5 ng cDNA and 500 nM forward and reverse primers, using a StepOne Plus machine (Applied Biosystems). The StepOne Plus protocol ran as 95° C for 2 minutes followed by 40 cycles of denaturation at 95° C for 3 seconds and annealing at a temperature 60° C for 30 seconds. Cycles-to-threshold (Ct) values were automatically obtained from the StepOne software v2.3. These values were exported to the Excel file and manually processed to generate fold change expression values. The expression of each gene of interest was normalized to the geometric mean of the expression of at least two housekeeping genes PPIA and RPL13A, generating the ΔC(t) value, and expression of 2^-ΔC(t)^ relative to the control.

Protein Extraction and Western Blotting

Media was gently aspirated and nanoneedle and FSW substrates were transferred to a new well plate on ice. Samples were rinsed two times with ice cold PBS. Cell lysate was extracted and pooled from 8 substrates for each sample in 300 µl of cell lysate extraction buffer that consisted of 4 M Urea (Ultrapure, Sigma-Aldrich), 150 mM NaCl (Sigma-Aldrich), PhosSTOP (Roche), and complete EDTA free protease inhibitor cocktail (Roche) by scraping within the well plate on ice. An immersion probe was used to sonicate lysates by applying a 10 second pulse at 200 W. Lysates were centrifuged at 15,000x*g* for 10 minutes at 4 ºC and supernatant was extracted. Qubit protein assay (Q33211, Thermo) was used to quantify protein extracted using fluorometric quantitation instrument Qubit 4 Fluorometer (Thermo). Lysates were prepared for Sodium Dodecyl Sulphate Polyacrylamide Gel Electrophoresis (SDS-PAGE) with 4x sample buffer containing β-mercaptoethanol in a 3:1 ratio and heated at 80 ºC for 5 minutes. Protein samples were separated on a gel by SDS-PAGE in TGS running buffer (Bio-rad) for 45 minutes at 100 V. The gel was transferred to a blot using Transblot-turbo (Bio-rad). Blots were probed with Cav-1 (1:1000), CLC (1:1000) and respective secondary antibodies (Li-Cor IR 680, IR 800, 1:1000) prepared in iBind solution (SLF1019, ThermoFisher Scientific) for fluorescent antibodies according the manufacturer's instructions. Immunoblotting was conducted using iBind Flex western device (ThermoFisher Scientific) according to the manufacturer's instructions. Blots were imaged using a Li-Cor Odyssey imaging system.

**Supplementary Figures**

­

Supplementary Figure 1 a) Schematic of hopping mode SICM for scanning cells on nanoneedles. In hopping mode SICM as used in this setup the probe approaches the sample from above thereby preventing the risk of collision particularly with high aspect ratio nanostructures such as our nanoneedles. Fixed samples are placed in a 35 mm dish in PBS. Voltage is applied between the electrodes in the pipette probe and in the bath producing an ion current. At every pixel, the pipette approaches the sample until a drop in current (I) of 0.25-1 % is achieved. At this point the height (z) is recorded and stored in a topographical map. The pipette is then retracted and moved to the next imaging location. b) 2D topographical maps of hMSCs on nanoneedles and FSW after 6 hours of culture. c) SEM images of hMSCs cultured on nanoneedles and FSW for 6 hours showing membrane ruffling. Scale bars = 5 μm.


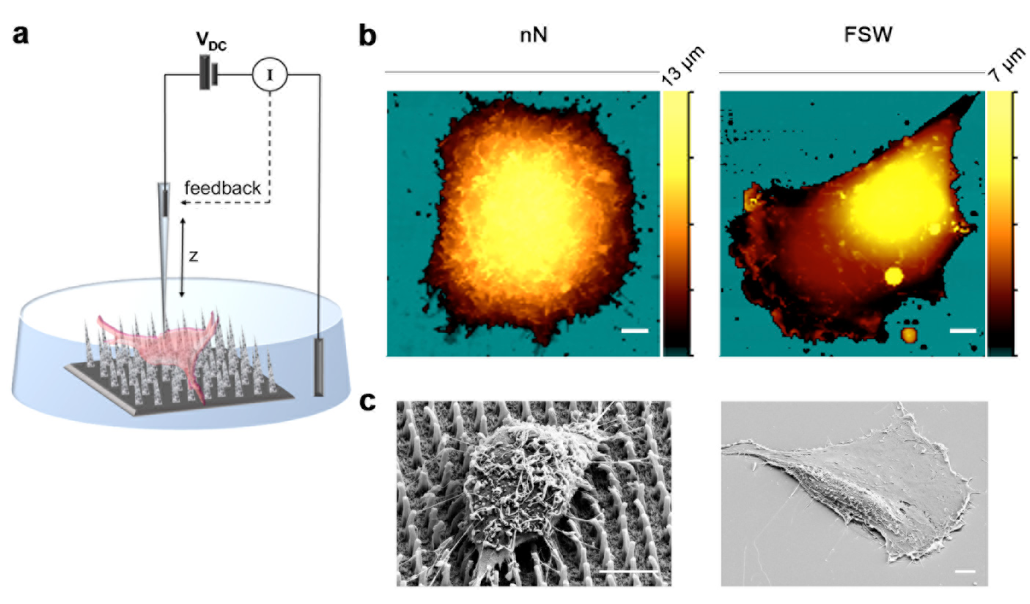


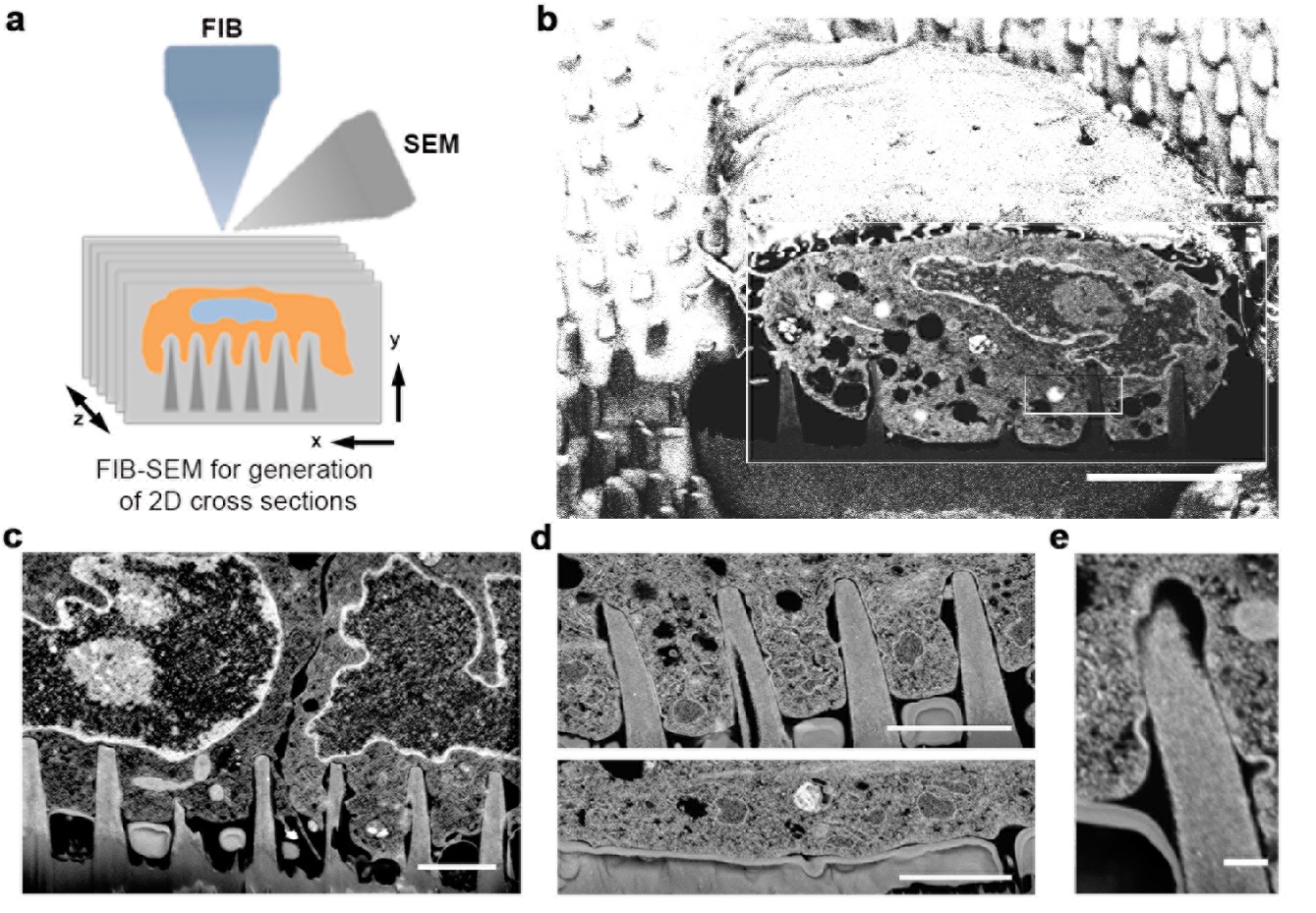


Supplementary Figure 2 a) Schematic of the FIB-SEM set up. 2D cross-sections of the cell-nanoneedle interface. 2D cross-sections of the cell-nanoneedle interface milled by the focused ion (gallium) beam at 30 nm intervals. Samples are milled orthogonally and the newly milled face is imaged using an SEM backscattered electron detector at the coincidence point (where the beams meet). b) Zoomed-out SEM image of the cross-section of an hMSC cultured on nanoneedles after 6 hours. Scale bar = 6 μm c) SEM images of a milled cross-section indicating two cells in contact while interfacing with nanoneedles. Scale bar = 2 μm. d) SEM images of milled cross sections of nanoneedle (top) and non-nanoneedle (bottom) regions indicating intact membranes. Scale bar = 2 μm. e) Zoomed-in view of the membrane-nanoneedle interface indicating membrane-wrapping and clathrin-like vesicles. Scale bar = 200 nm.


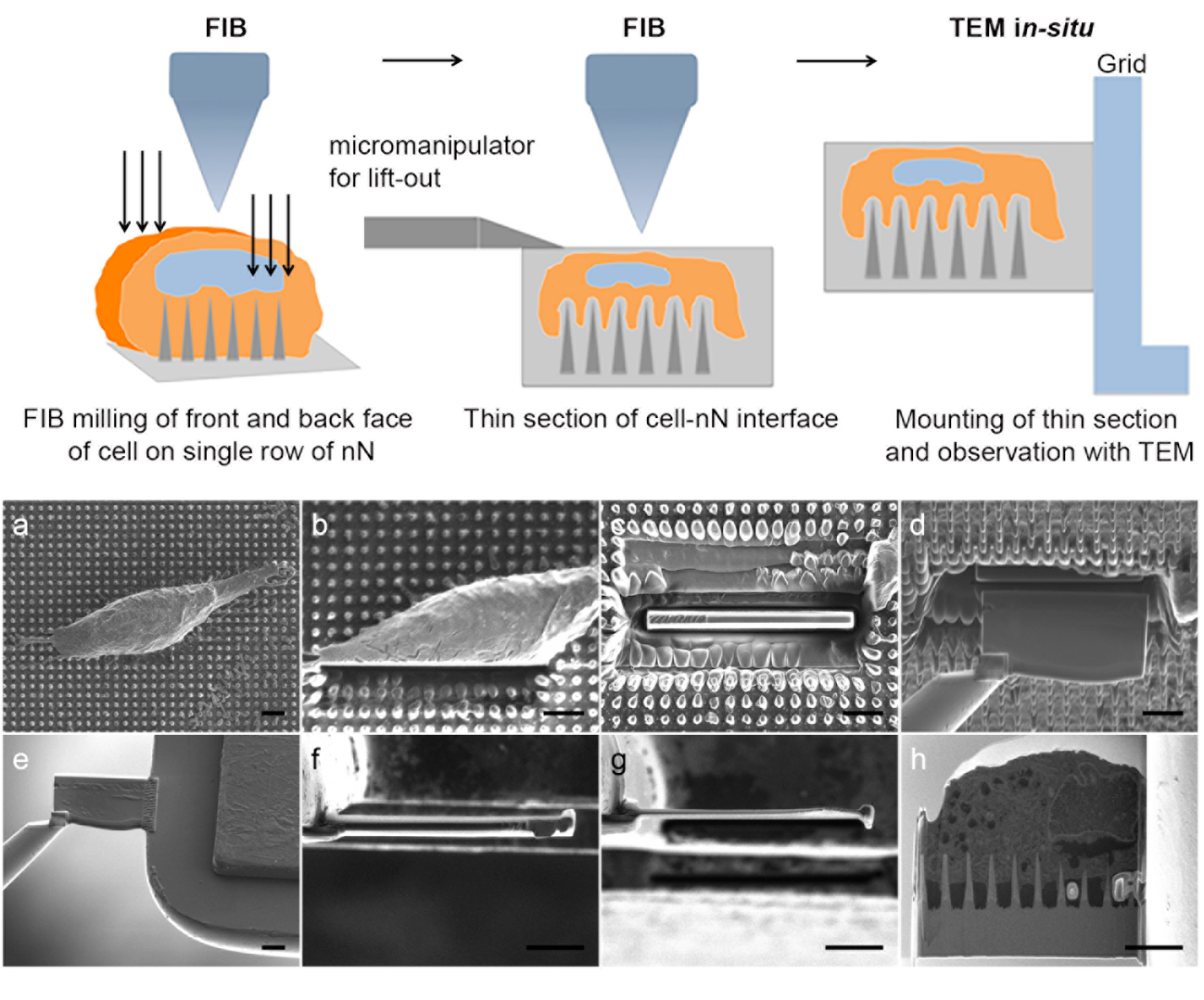


Supplementary Figure 3 Schematic and images of process flow for FIB lift out for generation of thin-sections. The sample (a) is milled at the front (b) and back (c) to generate a section of the cell with a single row of nanoneedles underneath it. d) A micro-manipulator is attached to the section using platinum deposition and the sample is milled below the base of the nanoneedles. e) The lift out section is mounted on a grid and the micromanipulator is released (f), after which the sample is thinned further (g) to desired thickness. h) the final front face of the thin section containing regions of interest prior to TEM imaging. Scale bars = 5 μm.

Supplementary Figure 4 a) Western blot of Caveolin-1, CLC and GAPDH in hMSCs after 6 hours of interfacing with nN or FSW. b) Quantification of western blot of Caveolin-1 and CLC normalized to GAPDH. N = 4 (CAV-1), N =1 (CLC), n = 8 (pooled samples). Data presented as mean ± S.D., p* < 0.05, two-tailed Mann-Whitney non-parametric test.

Supplementary Figure 5 a) Standard curve of Transferrin b) Quantities of Transferrin initially loaded on nanoneedle and FSW, and finally adsorbed on nanoneedle and FSW after 45 minutes of incubation. c) Standard curve of Cholera Toxin B subunit d) Quantities of Cholera Toxin B subunit initially loaded on nanoneedle and FSW, and finally adsorbed on nanoneedle and FSW after 45 minutes of incubation. e) Standard curve of Dextran 10 kDa. f) Quantities of Dextran 10 kDa initially loaded on nanoneedle and FSW, and finally adsorbed on nanoneedle and FSW after 45 minutes of incubation. Data shown as mean ± S.D., n = 3, n.s.= not significant (two-tailed Mann-Whitney test).





Supplementary Figure 6 a) Fluorescence microscopy images of hMSCs immunolabeled with rabbit anti-clathrin antibody, incubated with goat-anti rabbit Fab’ fragment followed by donkey anti-rabbit secondary AlexaFluor®488 and anti-rabbit secondary AlexaFluor®488 and b) the same with the addition anti-goat AlexaFluor® 555. Images indicate that primary rabbit immuno-label is converted to goat by goat anti-rabbit Fab’ fragment. Scale bars = 20 μm.





­


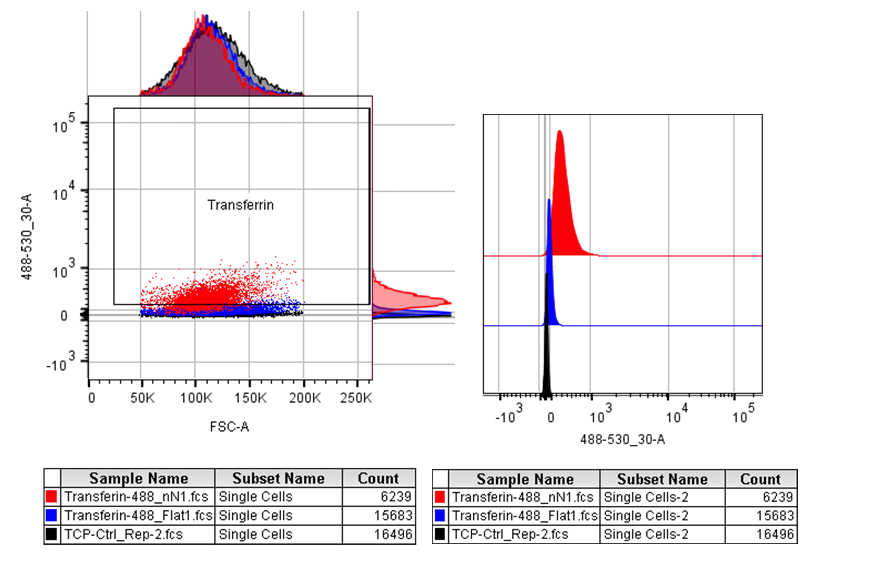


Supplementary Figure 7 Flow cytometry scatter plots and mean fluorescence intensity for clathrin-specific cargo Transferrin AlexaFluor®488 for hMSCs on cultured on nanoneedles (red), FSW (blue) and control TCP (no cargo, black) after 24 hours.


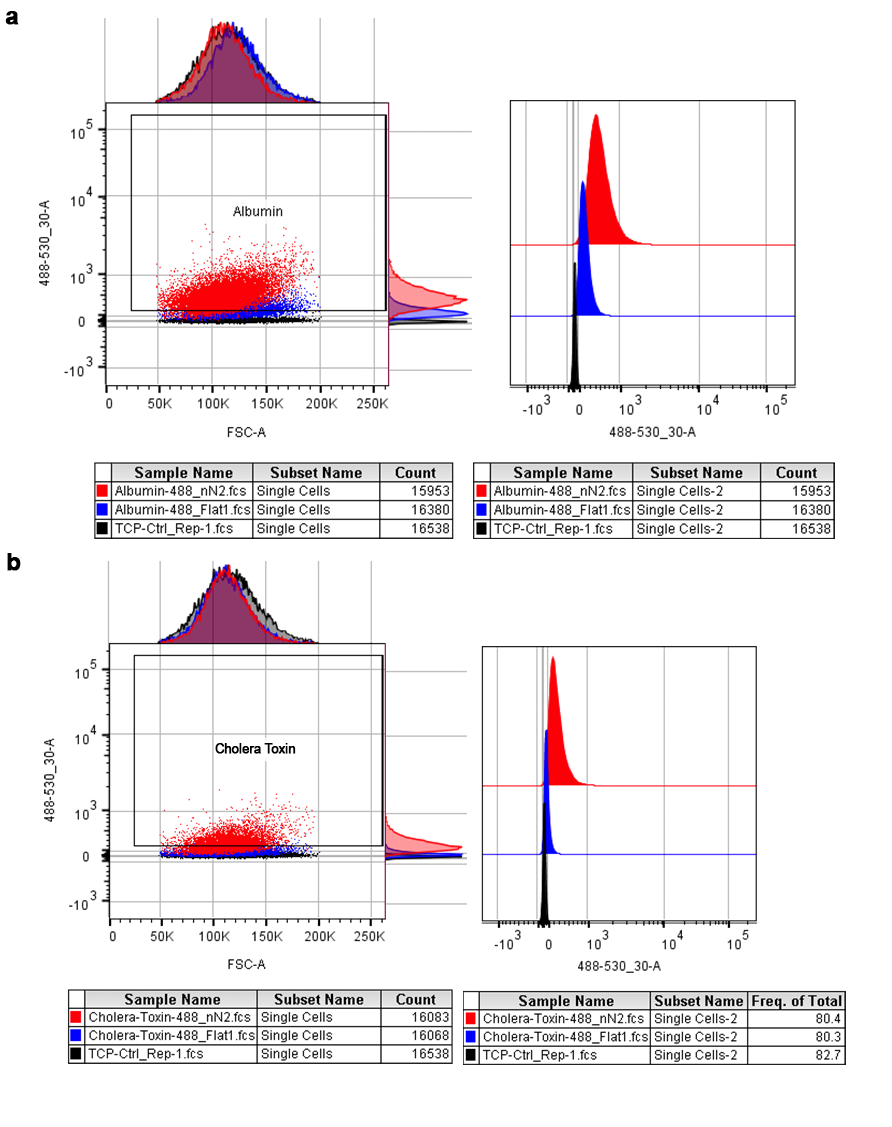


Supplementary Figure 8 Flow cytometry scatter plots and mean fluorescence intensity for caveolae-specific cargo Cholera toxin AlexaFluor®488 for hMSCs on cultured on nanoneedles (red), FSW (blue) and control TCP (no cargo, black) after 24 hours.


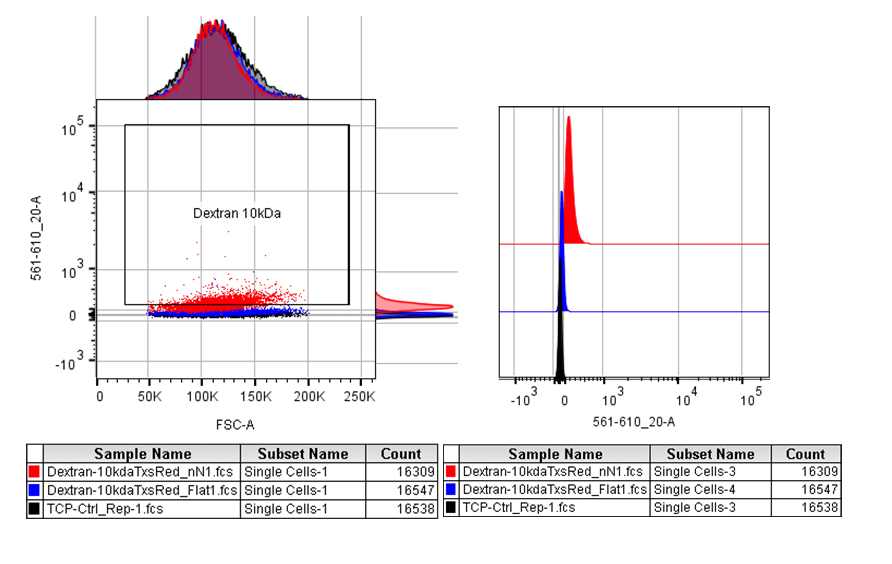


Supplementary Figure 9 Flow cytometry scatter plots and mean fluorescence intensity for micropinocytosis-specific cargo Dextran 10 kDa – TexasRed for hMSCs on cultured on nanoneedles (red), FSW (blue) and control TCP (no cargo, black) after 24 hours.


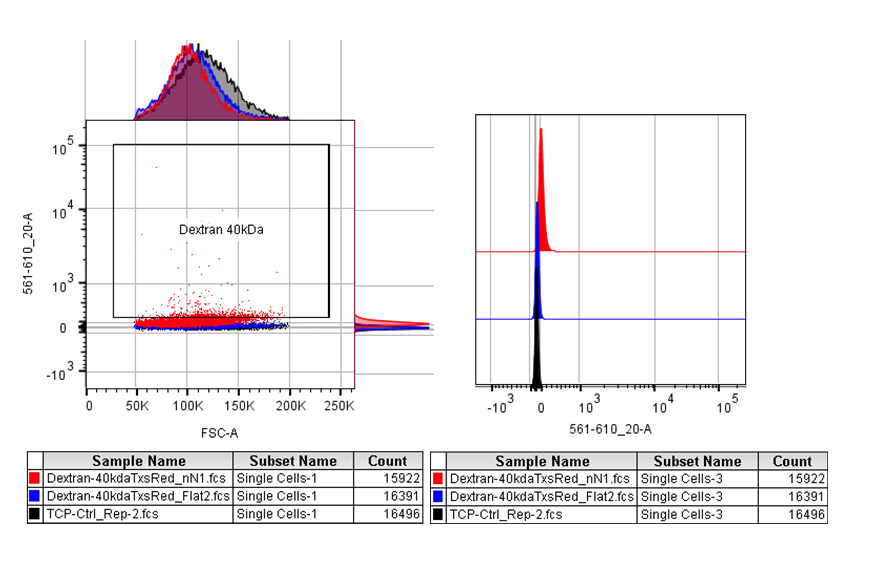


Supplementary Figure 10 Flow cytometry scatter plots and mean fluorescence intensity for micropinocytosis-specific cargo Dextran 40 kDa – TexasRed for hMSCs on cultured on nanoneedles (red), FSW (blue) and control TCP (no cargo, black) after 24 hours.


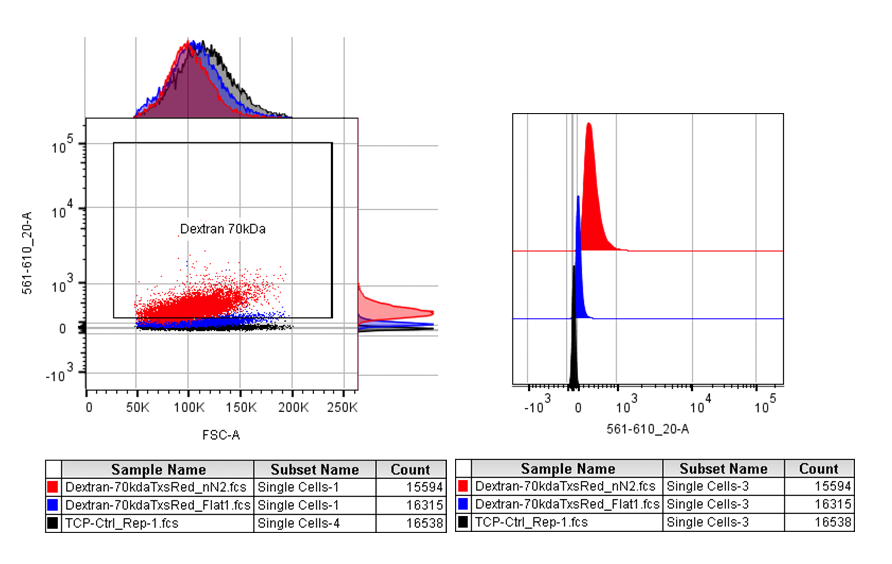


Supplementary Figure 11 Flow cytometry scatter plots and mean fluorescence intensity for micropinocytosis-specific cargo Dextran 70 kDa – TexasRed for hMSCs on cultured on nanoneedles (red), FSW (blue) and control TCP (no cargo, black) after 24 hours.

| **Experiment** | **GAPDH expression** | | **Difference between control and siRNA**  **(nN control – nN siRNA)** | **% Decrease (Difference/nN control *100)** |
| --- | --- | --- | --- | --- |
|  | **nN without siRNA delivery (control)** | **nN with siRNA delivery** |  |  |
| 1 | 0.72 | 0.29 | 0.41 | 59.47% |
| 2 | 0.84 | 0.57 | 0.68 | 32.19% |
| 3 | 1.44 | 0.89 | 0.62 | 37.90% |
|  |  | **Mean** | **0.57** | **43.19%** |
|  |  | **S.D.** | **0.14** | **14.38%** |

Table S1. Calculation of percentage decrease in expression of GAPDH per experimental replicate after nanoneedle-mediated delivery of GAPDH-siRNA. The difference between expression of GAPDH in control and siRNA treated samples in each experiment was used to calculate the percent change in expression compared to control. An average reduction in expression of 43% was achieved by siRNA-delivery via nanoneedle arrays.

| Gene Name | Accession Number | Forward primer (5’-3’)  Reverse primer (3’-5’) | Amplicon (bp) | Total primer concentration |
| --- | --- | --- | --- | --- |
| GAPDH | NM_002046.4 | GCACCGTCAAGGCTGAGAAC TGGTGAAGACGCCAGTGGA | 138 | 500 nM |
| PPIA | NM_021130 | CTTCACACGCCATAATGGC GTGATCTTCTTGCTGGTCTTG | 273 | 500 nM |
| RPL13A | NM_012423 | AAGTACCAGGCAGTGACAG CCTGTTTCCGTAGCCTCATG | 100 | 500 nM |

Table S2. qRT-PCR Primer Sequences

**References**

[1] C. Chiappini, E. De Rosa, J. O. Martinez, X. Liu, J. Steele, M. M. Stevens, E. Tasciotti, *Nat Mater* **2015**, *14*, 532.

[2] P. Novak, C. Li, A. I. Shevchuk, R. Stepanyan, M. Caldwell, S. Hughes, T. G. Smart, J. Gorelik, V. P. Ostanin, M. J. Lab, G. W. J. Moss, G. I. Frolenkov, D. Klenerman, Y. E. Korchev, *Nat Meth* **2009**, *6*, 279.
